# Successful Treatment of Lymphoplasmacytic Lymphoma/Waldenström Macroglobulinemia Complicated by Severe Autoimmune Neutropenia With Rituximab and Bendamustine

**DOI:** 10.7759/cureus.93392

**Published:** 2025-09-28

**Authors:** Taro Edahiro, Tetsumi Yoshida, Suzuka Nakatani, Hiroshi Ureshino, Tatsuo Ichinohe

**Affiliations:** 1 Department of Hematology and Oncology, Research Institute for Radiation Biology and Medicine, Hiroshima University, Hiroshima, JPN; 2 Division of Hematology, Respiratory Medicine and Oncology, Department of Internal Medicine, Faculty of Medicine, Saga University, Saga-city, JPN

**Keywords:** autoimmune neutropenia, bendamustine, lymphoplasmacytic lymphoma, rituximab, waldenström's macroglobulinemia

## Abstract

A 73-year-old woman was diagnosed with lymphoplasmacytic lymphoma/Waldenström macroglobulinemia (LPL/WM) with general fatigue and severe neutropenia. Although rituximab monotherapy was initiated, the neutrophil counts decreased within one week after rituximab administration. Subsequently, a combination of rituximab and bendamustine was initiated, and then her neutrophil counts became consistently within the normal range. The LPL/WM kept complete remission for two years. LPL/WM complicated with severe autoimmune neutropenia is rare. Rituximab and bendamustine brought strong lymphocyte depletion, leading to amelioration of autoimmune neutropenia. In this case, rituximab and bendamustine may have been active in patients with LPL/WM complicated with autoimmune disorders.

## Introduction

Lymphoplasmacytic lymphoma (LPL)/Waldenström macroglobulinemia (WM) is one of the mature B-cell neoplasms. Most cases of LPL/WM had immunoglobulin M (IgM) monoclonal gammopathy, which is generally associated with autoimmune reactions [1]. Mutation of MYD88 is common, and 95%-97% of LPL patients are positive. MYD88 mutation activates hematopoietic cell kinase that drives Bruton tyrosine kinase (BTK) prosurvival signaling. Recommended treatments are chemoimmunotherapies and BTK inhibitors. BTK inhibitors are preferred when patients have TP53 alterations [2]. It is well known that LPL/WM is often associated with autoimmune disorders, including autoimmune hemolytic anemia (AIHA) or immune thrombocytopenia (ITP). AIHA manifests as cold agglutinin disease, whose pathogenesis is binding IgM against I antigen on erythrocyte, leading to complement activation [3]. LPL/WM complicated with ITP is associated with both platelet-associated IgM (PA-IgM) and PA-IgG. Reduction of IgM may be effective against these LPL/WM-associated autoimmune disorders. However, severe autoimmune neutropenia with LPL/WM is a very rare condition, so optimal management of the disease condition has not been fully elucidated [1].

To date, three cases of LPL/WM with severe neutropenia have been reported. The first case, published in 1989, was an 88-year-old Japanese man. He was diagnosed with primary macroglobulinemia from a cervical lymph node biopsy, and the level of IgM was 3,200 mg/dL. He was administered prednisolone and vindesine, and his severe neutropenia temporarily improved. However, neutropenia relapsed because of myelosuppression from vindesine, and he died of septic shock. The second case, published in 2011, was a 54-year-old man. He was diagnosed with WM, and agranulocytosis progressed over two years from diagnosis. The level of IgM was 2,600 mg/dL. He was administered chlorambucil and rituximab. His neutrophil count gradually returned within four months after the therapy. The third case, published in 2018, was a 75-year-old woman. She was diagnosed with WM, and agranulocytosis progressed over eight years from diagnosis. The level of IgM was 3,000 mg/dL. She was administered rituximab, cyclophosphamide, and dexamethasone. Her neutrophil count recovered in three weeks after the therapy. She was continuously administered six cycles of rituximab and bendamustine, and her neutrophil count remained normal for two years [4-6]. We report a case of a patient with LPL/WM complicated with severe autoimmune neutropenia successfully treated with rituximab and bendamustine.

## Case presentation

**Figure 1:**
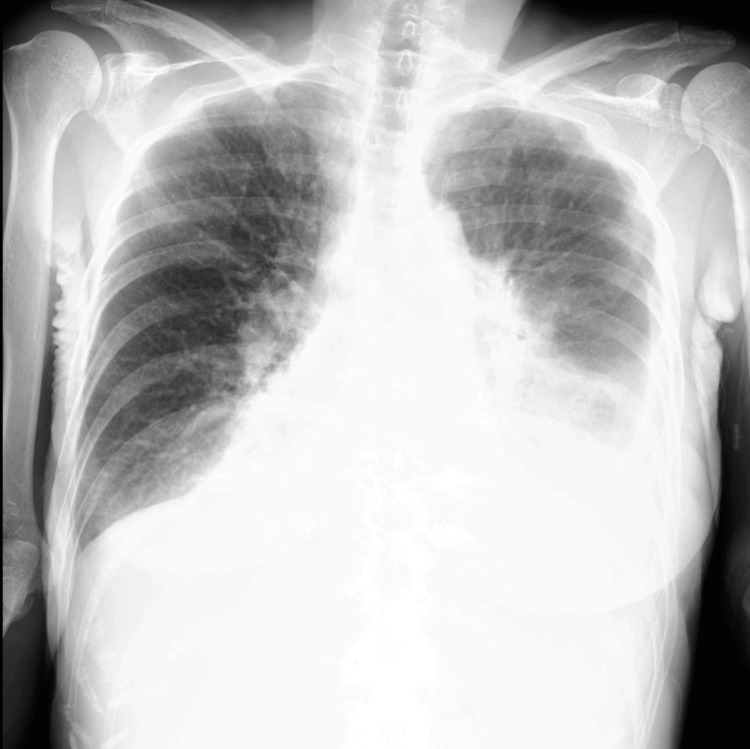
X-ray of the first visit X-ray showing obvious cardiac enlargement and pulmonary congestion because of chronic heart failure

A 73-year-old woman visited our hospital due to a one-month history of general fatigue and pancytopenia. Her comorbid diseases were chronic heart failure and lumbar compression fracture, and her past medical history was postoperative cataract. Her performance status was 3, and physical examination showed decreased breath sounds due to cardiac dilatation and bilateral pleural effusion (Figure 1). Her vital signs including body temperature, blood pressure, pulse rate, and consciousness were within the normal range, and saturation of percutaneous oxygen was decreased requiring oxygen supplementation (97% with nasal cannula 3 L). A complete blood count showed leukopenia with agranulocytosis (1.0 × 10^9^/L, with neutrophil, 0%; lymphocyte, 83%; monocyte, 17%), anemia (hemoglobin, 75 g/L), and thrombocytopenia (platelet count, 8.5 × 10^9^/L). Laboratory test showed elevated C-reactive protein (CRP, 6.83 mg/dL), soluble interleukin-2 receptor (8093 U/mL), ferritin (612.2 ng/mL), and monoclonal gammopathy of IgM (1647 mg/dL) with decreased IgG (970 mg/dL) and IgA (121 mg/dL), as shown in Table 1.

**Table 1:** Laboratory results before treatment, after rituximab monotherapy and rituximab and bendamustine treatment WBC: White blood cells; Ne: Neutrophil; H: Hemoglobin; PLT: Platelet; LDH: Lactate dehydrogenase; CRP: C-reactive protein; IgM: Immunoglobulin M; sIL-2R: Soluble interleukin-2 receptor.

	Before treatment	After rituximab monotherapy	After rituximab and bendamustine	Two years after the treatment	Normal range
WBC	1.0	1.6	2.8	3.7	3.3-8.6 × 10^9^/L
Ne	0	18.6	70.3	54.1	38.5%-81.5%
Hb	75	84	103	131	116-148 g/L
PLT	8.5	15.3	10.8	20	15.8-34.8 ×10^9^/L
LDH	169	79	173	149	124-222 U/L
CRP	6.83	3.85	0.08	0.08	0-0.14 mg/dL
IgM	1647	2162	10	14	50-269 mg/dL
sIL-2R	8093	8522	523	329	121-613 U/mL
κ/λ ratio	7.57	20.64	0.94	1.09	0.26-1.65

**Figure 2:**
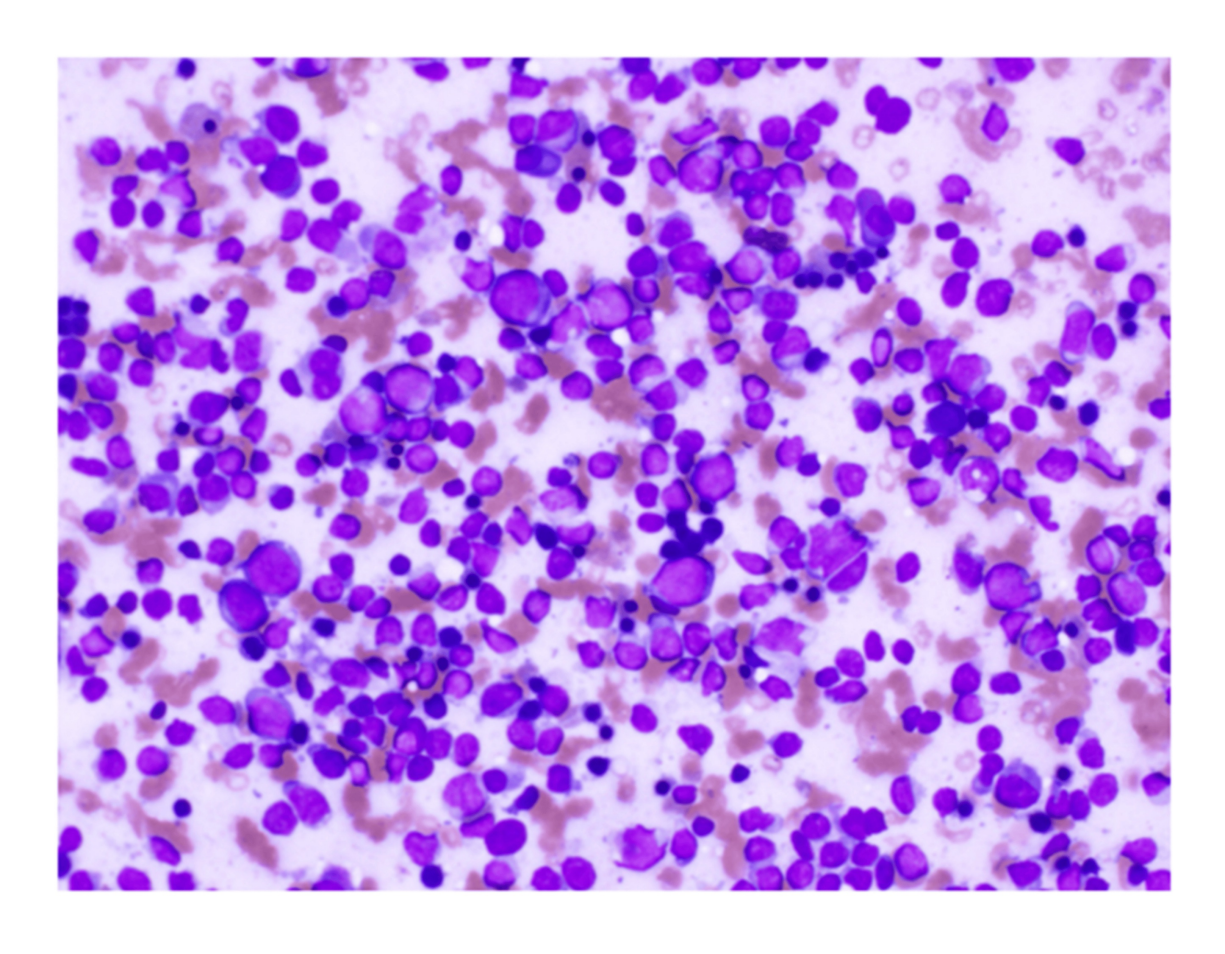
Bone marrow smear preparation Bone marrow aspiration revealed increased small lymphoid cells and decreased granulocytes (×40, May-Giemsa staining).

CH50 was slightly decreased (24 CH50/mL), and C3 and C4 were normal (121 and 18 mg/dL, respectively). Computed tomography (CT) did not show evident systemic lymphadenopathy and splenomegaly. Bone marrow aspirate revealed hypercellular with 10% of small-sized abnormal lymphocytes (suspected lymphoma cells) and severely decreased granulocytes (M/E ratio, 0.1), as shown in Figure 2.

**Figure 3:**
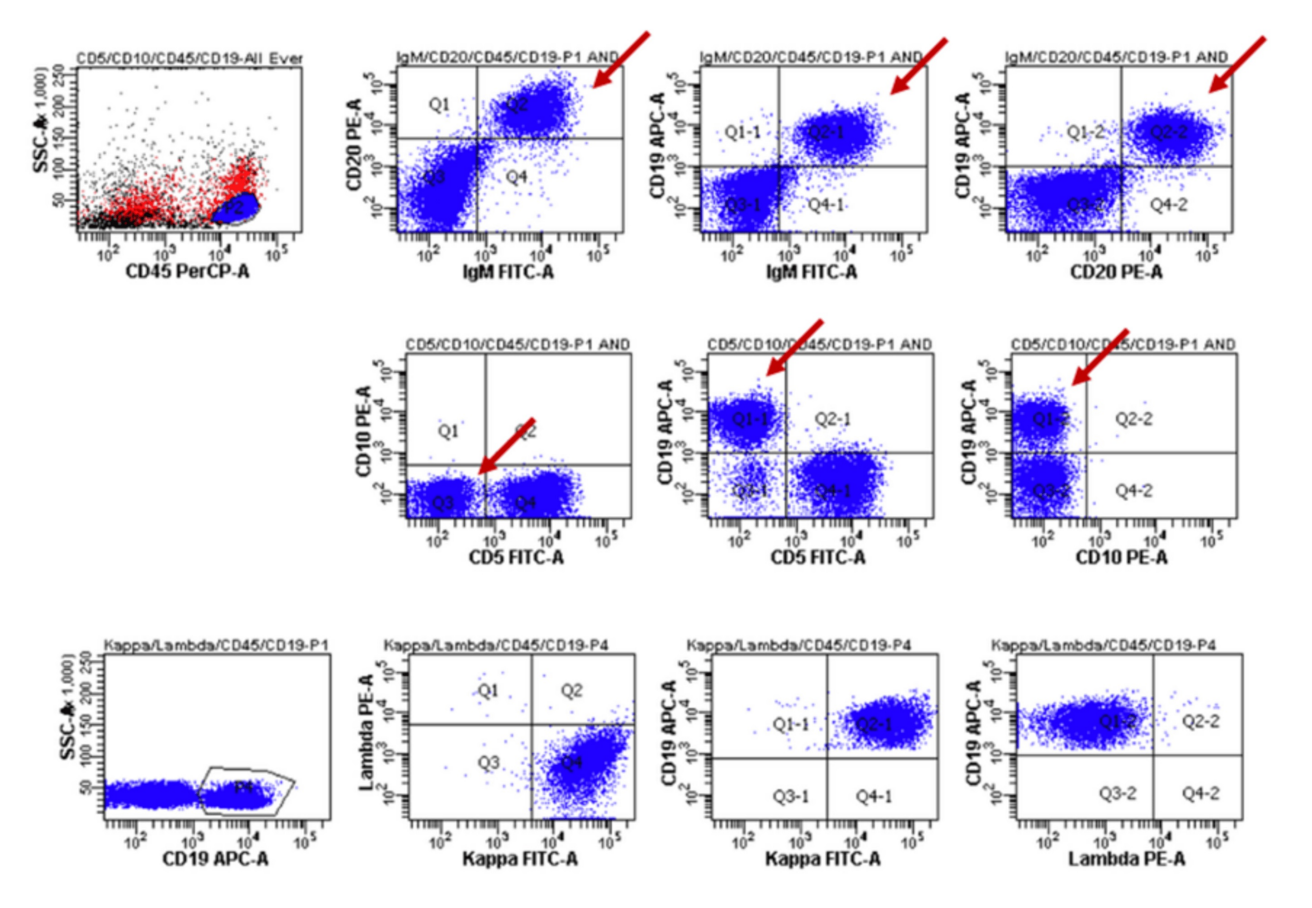
Flow cytometric analysis Abnormal lymphocytes were positive for CD19, CD20, IgM, and κ and negative for CD5, CD10, and λ.

**Figure 4:**
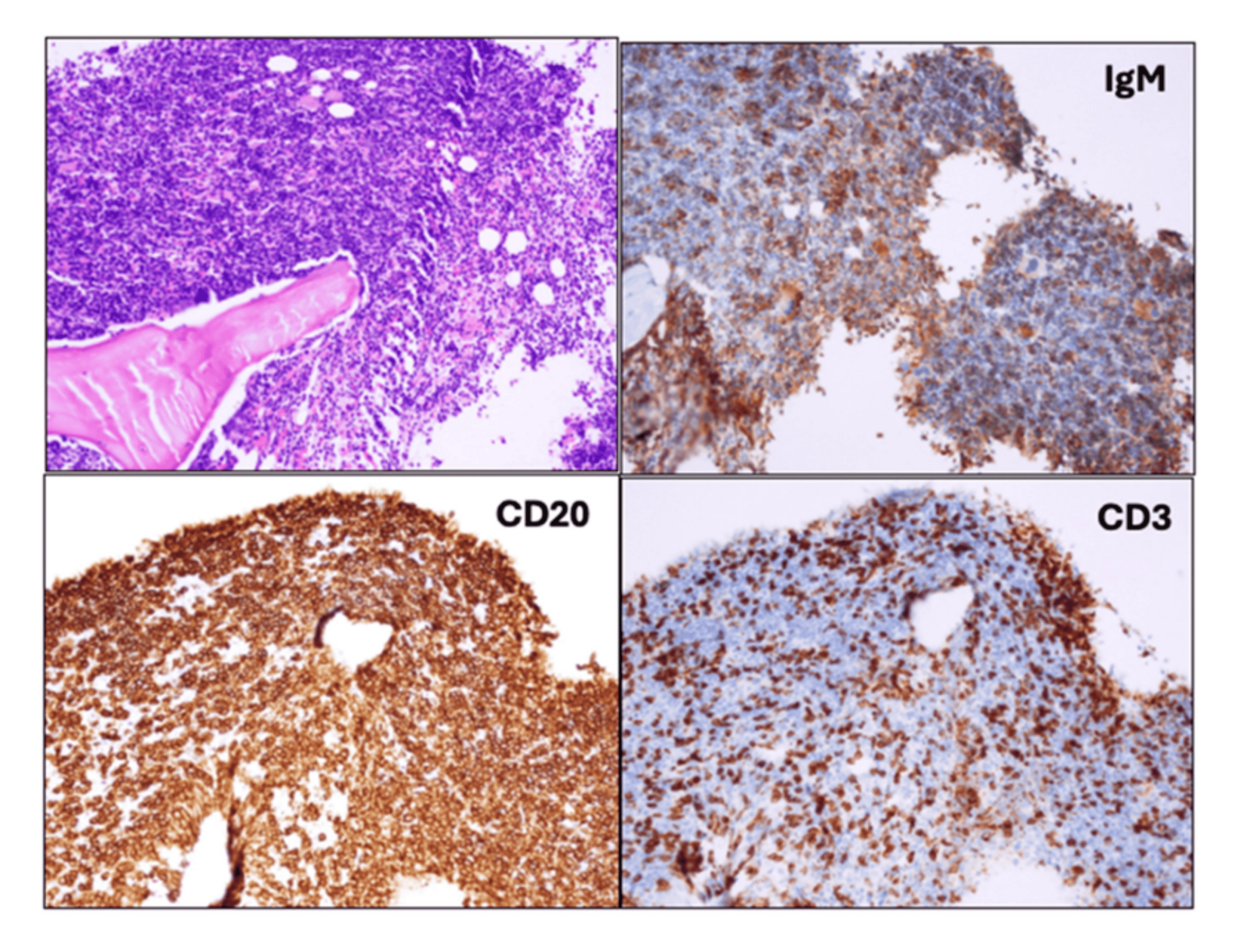
Bone marrow smear preparation The specimens showed increased lymphoid cells (hematoxylin-eosin stain), and they were positive for CD20 and IgM and negative for CD3 (×20).

Myeloblast was not increased (0.8%), and large granular lymphocyte lymphocytosis was not observed. Flow cytometric analysis revealed that abnormal lymphocytes were positive for CD19, CD20, IgM, and κ and negative for CD5, CD10, and λ (Figures 3, 4).

Fluorescence in situ hybridization analysis demonstrated the absence of TP53 deletions. MYD88L265P mutation analysis was not assessed because the mutation could not be measured easily in the clinical environment in Japan. Differential diagnoses were marginal zone lymphoma and chronic lymphocytic leukemia/small lymphocytic lymphoma [7], but they seemed negative because of the results of CT and flow cytometric analysis. The diagnosis of LPL/WM was made. Granulocyte immunofluorescence test for the presence of neutrophilic autoantibodies was negative.

**Figure 5:**
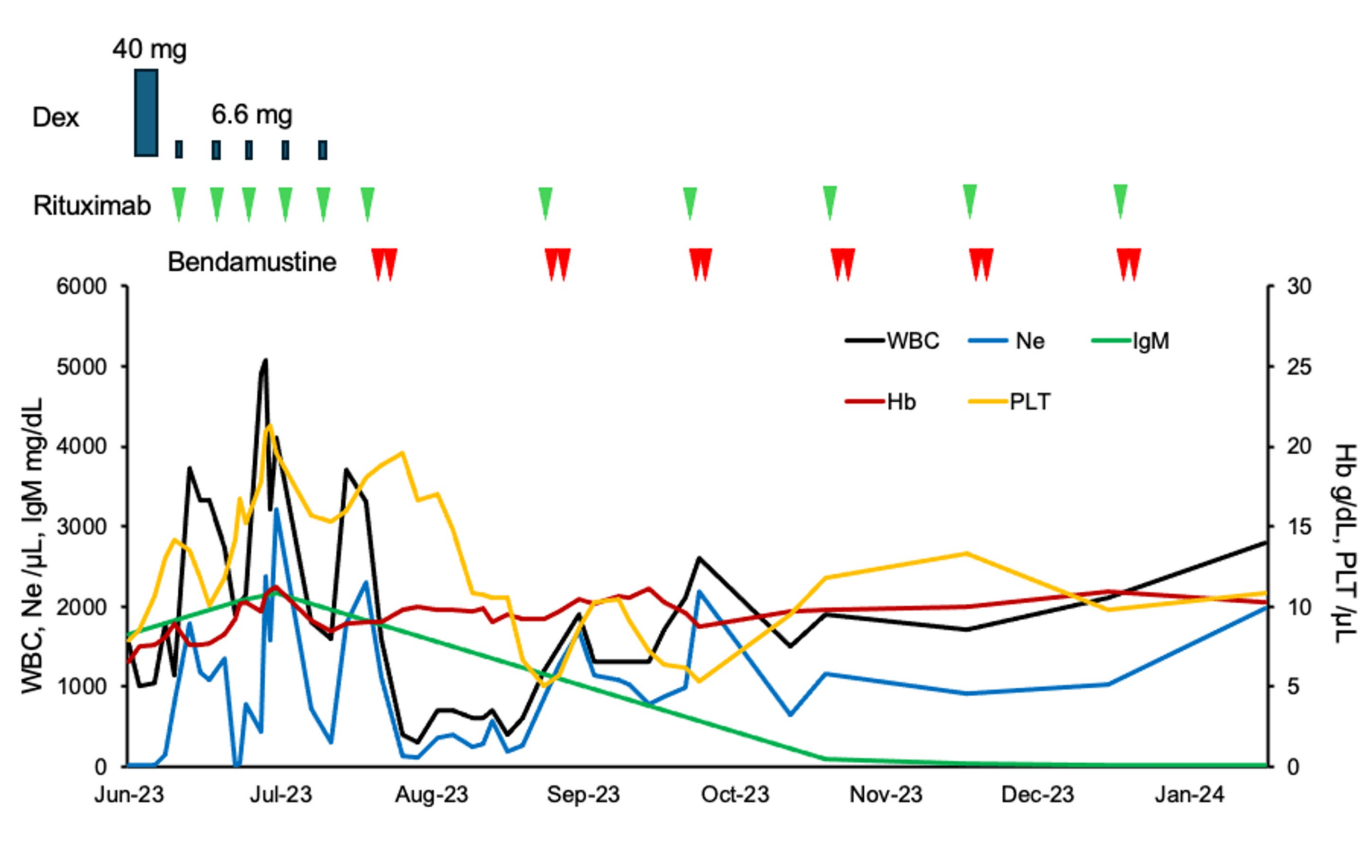
Clinical course Rituximab was administered weekly; however, neutrophil always started to decrease within one week. WBC: White blood cell; Ne: Neutrophil; IgM: Immunoglobulin M; Hb: Hemoglobin; PLT: Platelet.

She had severe heart failure in which cardiac wall motion was diffuse hypokinesis. At that time, we could initiate only ibrutinib as first-line treatment of Bruton’s kinase inhibitor, and we could also select rituximab monotherapy or rituximab in combination with chemotherapy in Japan. We avoided the use of Bruton’s kinase inhibitor as an initial treatment because of concerns about arrhythmia and cardiac strain as side effects of Bruton’s kinase inhibitor; thus, rituximab monotherapy was initiated. Then her neutrophil counts transiently returned to the normal range, while the neutrophil counts soon decreased. Rituximab monotherapy might be an insufficient treatment for the neutropenia, possibly involved in LPL/WM associated with autoimmune neutropenia. Rituximab and bendamustine were initiated, then neutrophil counts were soon increased corresponding to decreasing serum IgM level (Figure 5).

Six cycles of rituximab and bendamustine were completed without severe adverse events. No neutropenia or LPL/WM relapse occurred, and κ chain/λ light chain ratio of peripheral blood remained normal after about two years of rituximab and bendamustine treatment (Table 1).

## Discussion

LPL/WM is a rare B-cell neoplasm characterized by lymphoplasmacytic cell proliferation in the bone marrow with IgM monoclonal gammopathy. Patients with LPL/WM are often complicated with various autoimmune disorders, such as hemolytic anemia and immune thrombocytopenia [1].

To date, only three cases of LPL/WM with severe neutropenia have been reported. These cases were characterized by an aggressive clinical course with a relatively rapid increase in IgM levels, accompanied by a near-complete absence of neutrophils. The neutropenia was refractory to treatments such as intravenous immunoglobulin and/or granulocyte colony-stimulating factor, but following chemotherapy, both a reduction in IgM levels and an improvement in neutrophil counts were observed. Although antineutrophil antibodies could not be confirmed due to the profound neutropenia, the clinical course suggested an underlying autoimmune mechanism [4-6]. In the present case, the neutrophil count was also undetectable. Although the IgM level was not as elevated as in previously reported cases, the markedly elevated sIL-2R level suggests that the patient's WM was also thought to be of an aggressive subtype. Generally, the positive rate of antineutrophil antibody is about 50% in chronic idiopathic neutropenia patients [8]. In this case, antineutrophil antibodies were not detected; however, given the profound neutropenia, as in earlier reports, the absence of detectable autoantibodies may be attributable to the low neutrophil count. Notably, treatment with rituximab and bendamustine led to a reduction in IgM levels accompanied by recovery of neutrophil counts, supporting the possibility of an underlying autoimmune mechanism [9,10]. Bendamustine also exerts a profound immunosuppressive effect, particularly through marked suppression of T-cell populations. CD4 T-cells are activated by antigen presentation, and especially follicular helper T-cells promote antibody production of B-cells [11]. Therefore, not only B-cells but also T-cells may have an important role in the autoimmune mechanism [12,13]; therefore, bendamustine was beneficial for reducing pathogenic B-cells and T-cells.

The lower frequency of autoimmune neutropenia compared to AIHA and immune thrombocytopenia may be attributed to the following factors. IgM is known to efficiently activate the complement cascade, thereby promoting hemolysis and platelet destruction, particularly through recognition of erythrocyte surface antigens such as the I antigen [14] and platelet membrane glycoproteins [15]. Neutrophils are considered more resistant to complement-mediated damage, primarily due to their relatively elevated expression of regulatory proteins such as CD55 and CD59, in contrast to red blood cells and platelets [16]. Moreover, neutrophils have a short half-life in the circulation and rapidly migrate into tissues, which anatomically limits their exposure to circulating autoantibodies [17].

Neutropenia is commonly caused by bone marrow suppression due to chemotherapy, viral infections such as Epstein-Barr virus or cytomegalovirus, certain medications including antibiotics (e.g., trimethoprim-sulfamethoxazole), or underlying hematologic disorders such as large granular lymphocytic (LGL) leukemia [18]. These etiologies might not be considered contributory in this case.

## Conclusions

In conclusion, we encountered a case of LPL/WM with severe neutropenia that was successfully treated with rituximab and bendamustine. Myelosuppression of bendamustine was relatively mild, and recovery of neutrophil was fast; therefore, the risk of severe infection was low. This case suggests that rituximab plus bendamustine may be a viable treatment option for LPL/WM associated with autoimmune cytopenias, particularly in cases of severe neutropenia. This case report has limitations to generalizability because it is a single case, lacks antibody confirmation, and does not include the MYD88 mutation testing. Pay attention that the evidence of efficacy is insufficient; therefore, this treatment should be validated in more patients.
